# Y_2_O_3_ Nanoparticles and X-ray Radiation-Induced Effects in Melanoma Cells

**DOI:** 10.3390/molecules26113403

**Published:** 2021-06-04

**Authors:** Ioana Porosnicu, Cristian M. Butnaru, Ion Tiseanu, Elena Stancu, Cristian V. A. Munteanu, Bogdan I. Bita, Octavian G. Duliu, Felix Sima

**Affiliations:** 1 National Institute of Laser Plasma and Radiation Physics, P.O. Box MG-36, 76900 Bucharest-Magurele, Romania; ioana.porosnicu@inflpr.ro (I.P.); ion.tiseanu@inflpr.ro (I.T.); elena.stancu@inflpr.ro (E.S.); bita.bogdani@gmail.com (B.I.B.); 2 Faculty of Physics, Doctoral School on Physics, University of Bucharest, 405 Atomistilor Street, 077125 Magurele-Ilfov, Romania; o.g.duliu@gmail.com; 3 Institute of Biochemistry, Romanian Academy, 296 Splaiul Independentei, 060031 Bucharest, Romania; cristian.v.a.munteanu@gmail.com

**Keywords:** Y_2_O_3_ nanoparticles, A375 cell, X-ray irradiation

## Abstract

The innovative strategy of using nanoparticles in radiotherapy has become an exciting topic due to the possibility of simultaneously improving local efficiency of radiation in tumors and real-time monitoring of the delivered doses. Yttrium oxide (Y_2_O_3_) nanoparticles (NPs) are used in material science to prepare phosphors for various applications including X-ray induced photodynamic therapy and in situ nano-dosimetry, but few available reports only addressed the effect induced in cells by combined exposure to different doses of superficial X-ray radiation and nanoparticles. Herein, we analyzed changes induced in melanoma cells by exposure to different doses of X-ray radiation and various concentrations of Y_2_O_3_ NPs. By evaluation of cell mitochondrial activity and production of intracellular reactive oxygen species (ROS), we estimated that 2, 4, and 6 Gy X-ray radiation doses are visibly altering the cells by inducing ROS production with increasing the dose while at 6 Gy the mitochondrial activity is also affected. Separately, high-concentrated solutions of 25, 50, and 100 µg/mL Y_2_O_3_ NPs were also found to affect the cells by inducing ROS production with the increase of concentration. Additionally, the colony-forming units assay evidenced a rather synergic effect of NPs and radiation. By adding the NPs to cells before irradiation, a decrease of the number of proliferating cell colonies was observed with increase of X-ray dose. DNA damage was evidenced by quantifying the γ-H2AX foci for cells treated with Y_2_O_3_ NPs and exposed to superficial X-ray radiation. Proteomic profile confirmed that a combined effect of 50 µg/mL Y_2_O_3_ NPs and 6 Gy X-ray dose induced mitochondria alterations and DNA changes in melanoma cells.

## 1. Introduction

Rare earth oxide nanoparticles (NPs) have gained extensive attention over the last years due to their potential as nanodrugs for various biomedical applications including in vitro cancer cell imaging, in vivo tumor imaging through active targeting, X-ray imaging, photodynamic therapy etc. [1,2]. In particular, Europium (Eu) doped Y_2_O_3_ has been intensively studied as scintillator for medical imaging, radiation detection and dosimetry [3,4,5,6,7]. Given the excellent scintillating properties that have been reported for this material, Souris et al. [8] demonstrated that Y_2_O_3_:Eu NPs are promising materials for in situ, in vivo X-ray dosimetry. More recently, Kertzscher et al. analyzed a mixture of Y_2_O_3_:Eu and Yttrium orthovanadate doped with Europium (YVO_4_:Eu) as scintillating detector aimed at in vivo dosimetry for ^192^Ir brachytherapy [9]. Additionally, Chuang et al. showed that Y_2_O_3_:Eu covered by silica shell (Y_2_O_3_: Eu@SiO_2_) NPs act as independent photosensitizers for X-ray induced photodynamic therapy by generating an increased amount of reactive oxygen species (ROS) under X-ray irradiation that lead to cancer cell death [10]. This was the confirmation that Y_2_O_3_-based NPs may directly induce X-ray photodynamic effects without the presence of conventional photosensitizers.

Even though many applications of Y_2_O_3_-based NPs that involve radiation exposure have been reported, information regarding the effects induced by these NPs in cells under exposure to X-rays is missing. Previous studies regarding NPs cytotoxicity show that various sizes and morphologies can induce different biological responses [11]. As it concerns Y_2_O_3_, it has been demonstrated that smaller-size NPs are significantly more genotoxic than microparticles, and their surface chemistry can impact the cytotoxic response [12,13]. Nevertheless, for spherical Y_2_O_3_ NPs, cell viability was found invariant for various high concentrations, up to 500 µg/mL, with no statistical difference as compared with control samples [12]. A recent in vivo toxicity study of Y_2_O_3_ NPs shows that after intravenous injection in mice, the yttrium mostly accumulates in bones and causes cell apoptosis by breaking the intracellular phosphate balance in stromal cells [14]. On the other side, it has been reported that Y_2_O_3_ NPs have protective and antioxidant effect in murine hippocampal HT22 cells [15] and isolated rat pancreatic islets [16]. A significant free radical-scavenging capacity of Y_2_O_3_ NPs was observed in vivo for light-induced retinal damage on adult mice, and it has been suggested to be used in treating neurodegenerative disorders associated with ROS production, such as Alzheimer, Huntington, and Parkinson diseases [17,18]. However, the antioxidant effect was evidenced for low doses of Y_2_O_3_ NPs, up to 9 µM (about 20 µg/mL), which plateaued over this concentration [18]. Additionally, visible light exposure, having an energy much lower than X-ray, results in ROS production in cells due to the local heating effect while X-ray generates ROS due to ionization of oxygen containing molecules in cells. Therefore, the scavenging capacity of the Y_2_O_3_ could be limited for a certain degree of oxidative stress, nanoparticles concentration and even the cell type. An interesting in vitro result was revealed by Nagajyothi et al.’s study, which suggests that Y_2_O_3_ NPs exhibit anticancer activity inducing strong cytotoxic effect on renal carcinoma Caki-2 cells while showing nontoxic effect in normal MDCK cells [19].

Melanoma is a highly aggressive form of skin cancer and people suffering from this disease may undergo surgical resection of the tumor, chemotherapy, radiotherapy, or a combination of these treatments. Radiotherapy for melanoma treatment represents a second treatment modality being recommended for patients where surgery may result in unsightly scars or for patients which cannot undergo surgery due to age, comorbidities, or medication [20]. This is because melanoma has a wide range of sensitivity for exposure to radiation and presents a high risk of metastasis post radiation. Moreover, some in vitro studies indicate that melanoma cells present an intrinsic capacity of repairing sublethal DNA damages induced by radiotherapy [21] and its response to irradiation depends on tumor volume, dose and fraction size. In clinical practice, the radiation therapy for melanoma is prescribed at high dose per fraction (>4 Gy) but on adjuvant setting, more conventional fractionated doses are sometimes preferred [21]. Also, tumor metastasis represents a significant problem following surgery [22] and the possibility to increase the dose per fraction could overcome the radioresistance properties of human melanoma cell, thus allowing the use of the radiotherapy for melanoma treatment on a larger scale. Recent studies focused on using very high dose rate X-ray radiation by a plasma focus device to enhance sensitivity of A375 melanoma cell line [23].

The current study presents the biological response of human melanoma cells (A375) after treatment with different concentrations of commercially available Y_2_O_3_ NPs or exposure to various doses of superficial X-ray radiation. Our strategy to eventually improve the radiotherapy outcome relies on an X-ray photodynamic therapy induced by direct effect of Y_2_O_3_-based NPs for a precise and efficient treatment of tumors. For this, we have evaluated the intracellular production of reactive oxygen species, mitochondrial activity, colony-forming assay and DNA double-strand breaks generation. Additionally, the proteomic profile of A375 cells treated with nanoparticles and exposed to X-ray was analyzed to identify potential dysregulations of the signaling pathways involved in melanoma cells response to irradiation providing new potential therapeutic targets.

The main objective of the study was to systematically analyze the biological effects on A375 cells induced by either Y_2_O_3_ NPs or superficial X-ray radiation to eventually reveal the potential role of Y_2_O_3_ NPs during X-ray exposure. We believe these investigations are essential for biomedical cancer applications in the new field of theranostics where Y_2_O_3_ NPs could be used as intracellular dosimetric sensors during X-ray treatment via bioimaging of tumors [24,25,26,27].

## 2. Results

### 2.1. Size and Morphological Analysis of Y_2_O_3_ NPs

**Figure 1:**
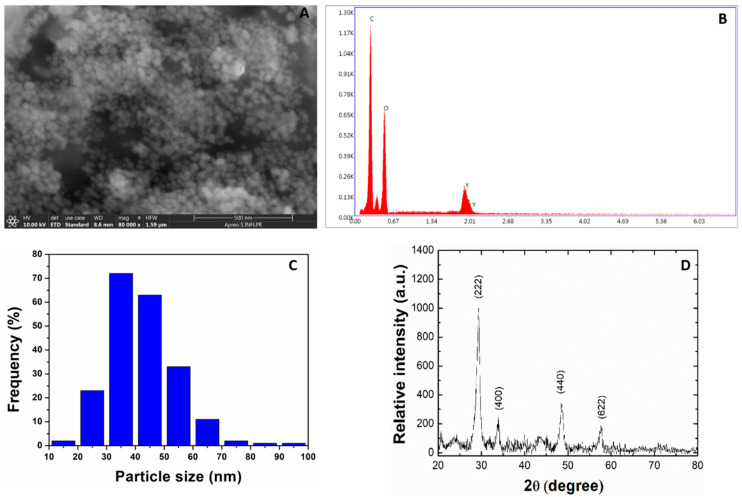
Y_2_O_3_ NPs characterization by (**A**) SEM analysis and corresponding (**B**) EDX pattern, (**C**) NPs size distribution from SEM images and (**D**) X-ray diffractograms.

Scanning electron microscopy (SEM) (Figure 1A) analysis has revealed spherical Y_2_O_3_ NPs particles with average size of 42.1 ± 11.8 nm (Figure 1C). The energy dispersive X-ray (EDX) spectroscopy investigations indicated pure, impurity-free, Y_2_O_3_ NPs supported by Y and O elements appeared in the recorded spectra (Figure 1B) with C element occurring due to the carbon tape used on sample holder. The crystallographic structure revealing the cubic phase of Y_2_O_3_ NPs was evidenced by XRD measurements (Figure 1D) while an average crystallite size of about 12 nm was determined by Scherrer’s formula applied to the most intense peak that corresponds to (222) plane.

### 2.2. Treatment with Y_2_O_3_ Generates ROS in Concentration-Dependent Manner

**Figure 2:**
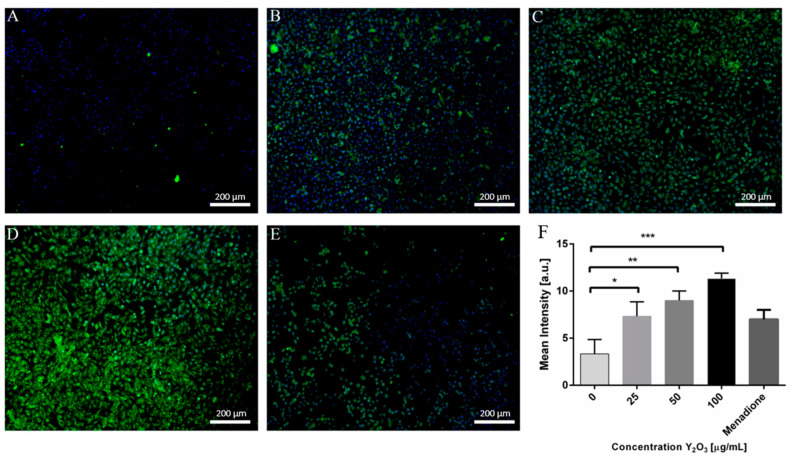
ROS generation by Y_2_O_3_ NPs treatment. A375 cells were plated on coverglass, left to adhere for 24 h and incubated for another 24 h with Y_2_O_3_ NPs. The cells were stained using a CellROX dye and observed under a fluorescence microscope. (**A**–**D**) ROS production generated by 0, 25, 50, 100 µg/mL NPs. (**E**) ROS induced by 100 µM menadione. (**F**) Quantification of mean ROS signal intensity (* *p* < 0.05, ** *p* < 0.005, *** *p* < 0.0005).

The subsequent analysis concerned the monitoring of ROS production in A375 melanoma cells treated with various Y_2_O_3_ NPs concentrations. To evaluate NPs uptake, the cells were stained for ROS detection, followed by fluorescence microscopy analysis (Figure 2A–D). As positive control for ROS generation cells were treated with menadione (Figure 2E) [28]. By quantifying the fluorescence signal (green) from the acquired images, one may observe a gradual increase of ROS production with the concentration of NPs (Figure 2F).

### 2.3. ROS Production in A375 Cancer Cells by X-ray Irradiation

**Figure 3:**
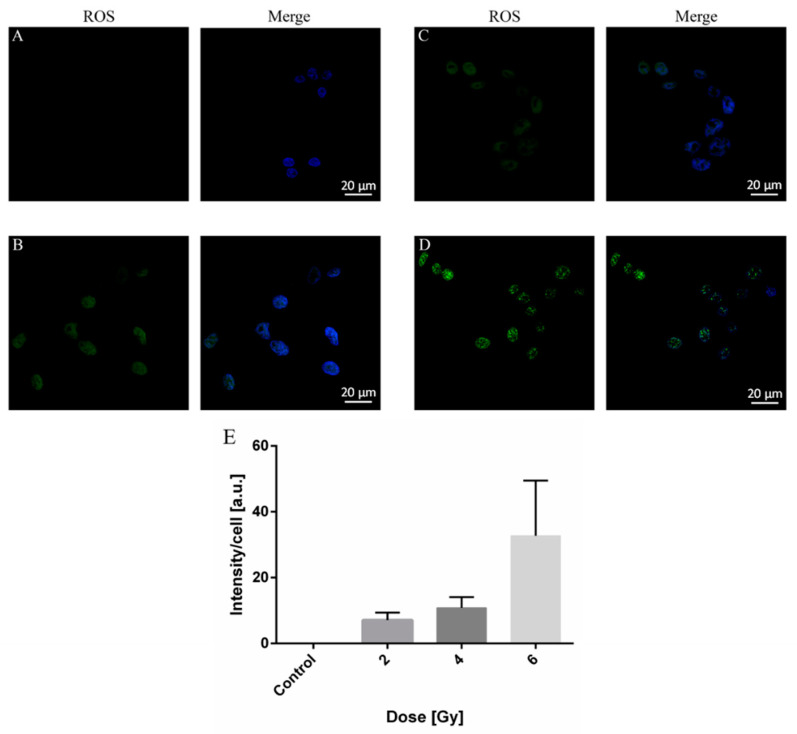
ROS generation in A375 cells induced by X-ray radiation: (**A**) not-exposed vs. exposed cells to (**B**) 2 Gy, (**C**) 4 Gy and (**D**) 6 Gy. (**E**) Quantification of ROS production from A–D by ImageJ.

The ROS generation induced by X-ray radiation of A375 cells was further estimated. We show in Figure 3 the fluorescence images of ROS generation in cells not exposed to radiation (Figure 3A) vs. cells exposed to 2, 4, and 6 Gy X-ray, respectively, doses (Figure 3B–D). The quantification of mean intensity signal from fluorescence images (Figure 3E) shows an increase in ROS production with the applied irradiation dose.

### 2.4. Radiation-Induced Modifications of A375 Cancer Cell Mitochondria Activity

**Figure 4:**
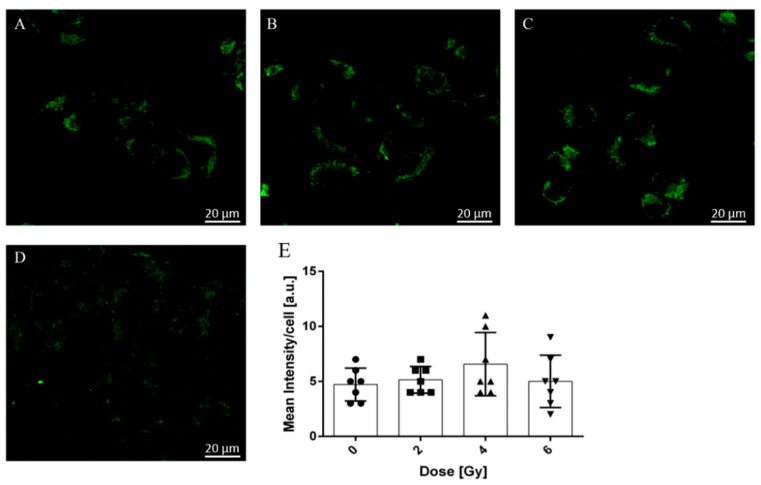
Mitochondrial morphological changes in A375 cells after X-ray radiation: (**A**) not-exposed (●) vs. exposed cells to (**B**) 2 Gy (■), (**C**) 4 Gy (**▲**) and (**D**) 6 Gy (**▼**). (**E**) Quantification of each cell fluorescence from A–D by ImageJ (the number of symbols for each dose represents the number of cells).

We further evaluated cell mitochondria after exposure to X-ray radiation. As shown in Figure 4, no significant changes in mitochondrial activity could be noticed for the 2 and 4 Gy doses, respectively, (Figure 4B,C) as compared to not-exposed samples (Figure 4A). However, in the case of cells exposed to 6 Gy dose of irradiation (Figure 4D) the potential of the mitochondria was considerably reduced suggesting the mitochondria functioning is likely affected. These findings are supported by the quantification of fluorescence intensity for each image (Figure 4E), which showed a rather slight increase for the samples exposed to 2 and 4 Gy X-ray dose and a reduction for sample exposed to 6 Gy, respectively. Also, we did not observe significant morphological changes for the 2 and 4 Gy treated sample compared to the control. However, in the case of cells exposed to 6 Gy dose of irradiation, it can be stated that the integrity of the mitochondria is heavily affected.

### 2.5. Combined Effect of NPs and Radiation Induced DNA Damage in A375 Cancer Cells

**Figure 5:**
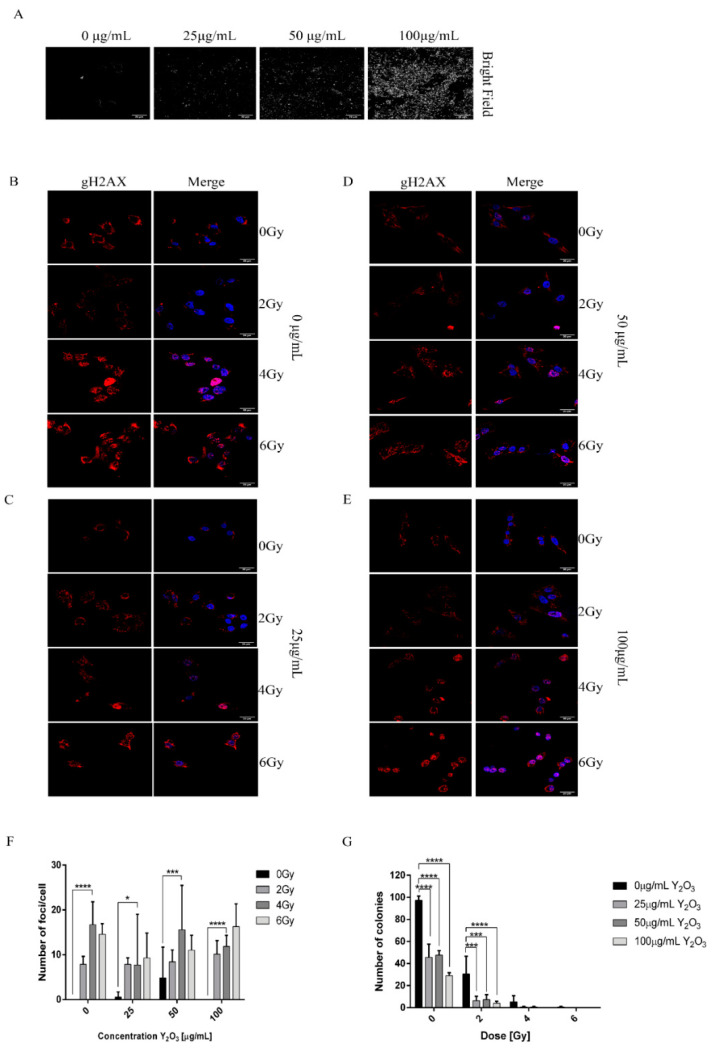
DNA damage induced by X-ray radiation. (**A**) Bright field image depicting the uptake of different concentrations of NPs (**B**–**E**) A375 cells treated with different concentrations of NPs and irradiated with 0, 2, 4, 6 Gy and stained for γ-H2AX. (**F**) Quantification of number of foci/cell using ImageJ software. (**G**) Clonogenic assay of samples treated with 0, 25, 50, 100 μg/mL Y_2_O_3_ and exposed to 0, 2, 4, 6 Gy doses of radiation (* *p* < 0.05, *** *p* < 0.005, **** *p* <0.0005). The scale bars in Figs (**A**–**E**) are of 20 μm.

A common effect of ionizing radiation on cells exposed to this type of radiation is the induction of DNA damage, more specifically double-strand DNA breaks. We first qualitatively verified that the cell uptake of NPs for 24 h corresponds to various concentrations of NPs used with the initial solutions. This allowed us to estimate radiation effect combined with NPs concentrations. As depicted in Figure 5A (bright field panel), the density of NPs observed intracellularly increases with NPs concentration. By quantifying the number of foci in Figure 5B–E for each irradiated sample one may estimate that in some cases the Y_2_O_3_ NPs do alter the X-ray effect on melanoma cells In particular, one may observe a rather increase of number of foci for the same Y_2_O_3_ NPs concentration with increasing dose exposure. This effect is also visible when increasing the Y_2_O_3_ NP concentration from 25 to 100 µg/mL. On the other hand, at 4 Gy X ray dose an increase of foci number was found with increase of Y_2_O_3_ NPs from 25 to 50 µg/mL, followed by a decrease when concentration became 100 µg/mL. Although ROS generation in A375 cells increased with applied X-ray dose or Y_2_O_3_ NPs concentration, this tendency was not always kept for double-strand DNA breaks in irradiated cell samples containing NPs.

A colony-forming assay (Figure 5G and Figure S1) evidenced a rather synergic effect of nanoparticles and radiation on radiotherapy enhancement. By adding the NPs to cells before irradiation, one may clearly observe decreasing the number of proliferating colonies-generating cells.

### 2.6. Proteomic Profile of A375 Cancer Cells Exposed to Y_2_O_3_ NPs X-ray

**Figure 6:**
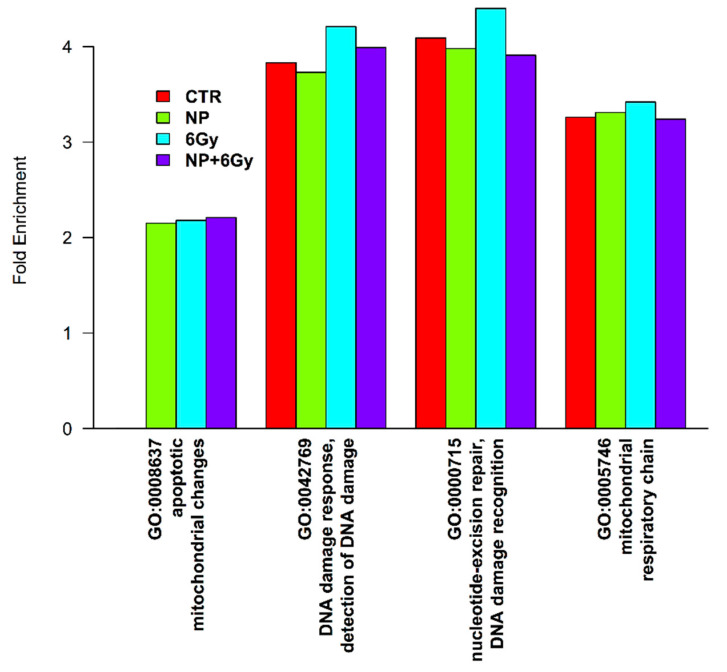
Gene Ontology analysis of the identified proteins. Fold enrichment, relative to the human proteome background, of GO terms related to DNA damage response and mitochondrial changes for all four conditions analyzed: control sample (CTR), treated with nanoparticles (NP), irradiated (6 Gy) and treated with nanoparticles and irradiated (NP + 6Gy).

The molecular pathways involved in the cell’s response to X-ray radiation were analyzed from the proteomic profiling obtained by mass spectrometry. For this, A375 cancer cells were cultured for 24 h with Y_2_O_3_ NPs of 50 μg/mL concentration and half of the cell samples were subjected to a 6 Gy X-ray dose. As positive controls, we have used Y_2_O_3_ NPs-free A375 cell samples irradiated with the same X-ray dose. We chose these conditions as the irradiation dose is high enough to produce molecular modifications while the concentration of Y_2_O_3_ NPs is relevant for eventually changing the radiation effect. Among the 4000 identified proteins, we searched for significant enriched pathways using the DAVID bioinformatics resource [29,30]. The identified proteins were annotated using Gene Ontology (GO) key terms. As compared with the human proteome, we have observed higher fold changes for proteins involved in mitochondrial respiratory chain related to the mitochondria apoptotic changes for cell samples irradiated with 6Gy dose (Figure 6). In addition, key proteins involved in DNA damage response mechanism were found considerably upregulated, in particular, specific proteins with role in DNA damage recognition and repair. Interestingly, cell samples exposed to the same irradiation dose in the presence of Y_2_O_3_ NPs were found less enriched, almost like unexposed cell sample (Figure 6). These observations may suggest that treatment of A375 melanoma cells with Y_2_O_3_ NPs does alter these response pathways to X-ray irradiation.

One of the master regulators of sensing DNA double-strand breaks induced by ionizing radiation is ATM (ataxia-telangiectasia mutated) [31]. This protein activates by phosphorylation regulators of cell cycle arrest. One of the transducers CHK1 (checkpoint kinase-1) acts downstream of ATM [32] and is identified in our proteomic profiling. CHK-1 is responsible, among others, for the phosphorylation of p53 (tumor suppressor protein) a central stress protein in the DNA damage response. Phosphorylation of p53 at different sites [33] results in dissociation from E3 ubiquitin-protein ligase Mdm2 and accumulation in the nucleus, where it acts as a transcription factor [34]. The amount of DNA damage and the number of modifications of p53 determine which of the two pathways are activated by p53, cell survival or cell death [35]. Additionally, the decision between cell cycle arrest and apoptosis is determined by the number of p53 responsive genes in different lineages [36]. The cell cycle arrest is induced by the activated form of p53 by phosphorylating p21 (CDKN1A, cyclin-dependent kinase inhibitor 1A), which in return blocks the activity of CDK4 and CDK6, forcing the cell to enter G1 arrest [37]. Furthermore, if the transcription of p21 is high it can also block cell cycle transition from G2 to M phase by binding to the complex formed between CDK1-cyclinB [38]. Our data show that p21 protein is found in unexposed cell sample and those treated with Y_2_O_3_ NPs, but not for cell samples that have been exposed to radiation only. Moreover, the peptide spectrum match (PSM), which shows the frequency of the peptides in the sample [39] of CDK1, CDK4 and CDK6 are higher for cells irradiated in the presence of Y_2_O_3_ NPs compared to cell irradiated without NPs, suggesting that Y_2_O_3_ might play a role in alleviating the effect of DNA damage induced by X-ray irradiation.

The next step in DNA damage response, after cell cycle arrest, is to repair the double strand breaks (DSB). This step can be achieved mainly by two pathways, namely non-homologous end joining (NHEJ) and homologous recombination (HR) [40]. NHJE acts by repairing simple DSB with complementary overhangs and phosphorylated and hydroxylated 5′ and 3′ ends. Its drawback are if DNA ends require processing before ligation the process will become inaccurate, causing short additions or deletions in the DNA sequence, translocation or rearrangements of chromosomes [41]. The HR pathway requires an undamaged sister chromatid for recombination and acts in the G2 or late S phases.

Our findings suggest that the repair pathways of DNA damage double-strand breaks, seem to be active for cell irradiated samples to different extents [40]. Based on the identified PSMs proteins distribution across the samples the NHJE pathway (Non homologous joint ending) is upregulated compared to HR (homologous recombination) (Table 1). We have also identified repair cross-complementing protein 5 and 6 (XRCC5 and XRCC6) to be highly expressed. These two proteins form a heterodimer involved in a repair pathway of DNA followed by binding at the 5′ end, thus blocking the HR pathway [41] and this process is strengthened by activating the p53 binding protein 1 (53BP1). The HR pathway is initiated by MRE11, RAD50, and NBS1 a complex termed MRN [42] (Inhibiting homologous recombination for cancer therapy). For this pathway, we detected MRE11, RAD50 and CTIP.

In conclusion, the proteomic profile suggest that two main pathways are involved in the molecular response to the X-ray irradiation of A 375 cancer cells: (i) the DNA damage repair pathway, showing that a 6 Gy dose induces DNA changes and (ii) the mitochondrial respiratory chain which induces mitochondria alterations.

## 3. Discussion

Radiotherapy is considered a standard tumor treatment. Nevertheless, recurrence is often encountered and radioresistance may appear, all these are reflected in a low survival rates for cancer patients [43]. Melanoma is a very aggressive form of cancer known to be resistant to radiation; however, it also exhibits a radiosensitive phenotype found in a significant number of cell lines [44]. Novel approaches to improve radiotherapy by nanotechnology are now attempted to overcome radioresistance of melanoma tumors [45,46]. Indeed, various nanomaterials are known to induce cell apoptosis by mitochondrial injury [27]. Relevant biochemical events involve altered energy metabolism, mitochondrial outer membrane permeability, mitochondrial swelling, release of pro-apoptotic BCl-2 family proteins and loss of mitochondrial inner membrane potential, which eventually could decide cell fate [27]. A promising strategy to enhance the biological effect of radiotherapy refers to the NPs induced sensitization of tumor cells without affecting normal tissue cells. The studies have been mostly concentrated on low-energy radiation and high atomic number (Z) materials, in which the effect is enhanced since such materials are known to absorb keV X-rays, while radiosensitizing effect of MV X-rays may open new perspective since it is not attributed to high-Z material alone. Indeed, the use of conventional high-Z NPs for radiosensitization is due to strong photoelectric effect of keV photons; however, some studies also revealed that sensitization is possible on B16F10 melanoma cells with 6 MV radiation sources, most probably due to other involved biological mechanisms [47].

On the other hand, NPs alone are causing oxidative stress in cancer cells besides irradiation [48]. The NPs-assisted sensitization is the main factor responsible for ROS production increase due to the dissociation of water by enhancing localized emission of secondary electrons from NPs. An alternative mechanism responsible for ROS increase could be considered the alteration of local chemical composition. In case of oxide NPs, oxygen dissolution could generate ROS while, at the same time, any dissolved metal ions may also enhance ROS by redox activity. This may change the radiobiological response either by ROS enhancement or induced toxicity. Compound solubility generally corresponds to a pH decrease and then it becomes difficult to identify the various roles of NPs and resulted solution [49]. On the other hand, different cell lines exhibit totally different behavior when exposed to the same NPs stimuli [50] while, in the same time, the uptake of NPs is cell dependent [51]. ROS was measured in many sensitization studies, without offering a complete understanding of the NPs effects with respect to chemical processes and biochemistry.

Alternatively, photosensitizer-independent X-ray photodynamic therapy using Y_2_O_3_ based NPs could be of interest for precision therapeutic treatments of deep-seated tumors [10]. Our experiments of irradiation with superficial X-ray sources used in radiotherapy for skin cancer were found to induce cellular damage via ROS generation. Additionally, the Bremsstrahlung radiation generated at a tube potential up to 100 kV presents a maximum of intensity that overlaps the K-edge binding energy of Y_2_O_3_ (at 17.04 keV) (Figure S2). We predict that this could maximize the generation of photoelectrons and scattered electrons that may further ionize the oxygen containing molecules from cell and generate ROS around nanoparticles. Also, keV photons deliver significant part of their energy in the first 5 mm layers of tissue and increasing the beam energy could lead to unnecessary exposure of healthy tissue under the skin tumor [52].

Our first aim was to evaluate the mitochondrial activity and reactive oxygen species production [53]. The experiments suggested that uptake of Y_2_O_3_ NPs promotes ROS generation with increase of concentration. However, the cells kept good viability, independent on Y_2_O_3_ NP concentration, in agreement with other studies (Figure S3) [12]. Accumulation of NPs causes further loss of cell proliferative capacity under different irradiation doses, probably due to redox signaling that upregulates growth factor pathways to sustain growth and proliferation of cancer cell [54]. This hyper-metabolism may trigger generation of ROS in cancer cells [54]. Since the Y_2_O_3_ NPs could perturb the equilibrium between ROS and antioxidants due to its direct antioxidant activity, ROS increase may target the nuclear factor kappa-light-chain-enhancer of activated B cells (NF-κ B), known to control cell survival of tumor cells [55,56]. These findings are in agreement with other studies that report cytotoxicity and genotoxicity by generation of ROS in human embryonic kidney cells and human foreskin fibroblasts [12,57]. 

Further results suggested that by increasing irradiation dose at 6 Gy it can affect the cell capacity to grow, while the morphology of mitochondria is severely affected. Indeed, it was also demonstrated that at low X-ray energy, gold NPs radiosensitising effect can induce DNA damage, with mitochondria driving the NPs radiosensitization [58].

In next experiments, we found that the colony-forming assay rather supports the synergic effect of NPs and radiation with eventual impact on radiotherapy enhancement. By adding the NPs to cells before irradiation, one may clearly observe decreasing the number of proliferating colonies-generating cells, most probably due to cell apoptosis [10]. Indeed, cell apoptosis may be induced by either mitochondrial injury we have observed at 6 Gy irradiation or DNA damage. In our experiments, an increase of number of foci was evidenced for a given Y_2_O_3_ NPs concentration with increasing the X-ray dose. In addition, this effect is enhanced with NP concentration from 25 to 100 µg/mL. On the other hand, we have observed a lower number of γ-H2AX foci exhibited by cells upon exposure to the combined effect of NPs and radiation as compared with cells exposed radiation only. We hypothesize that this result may be the consequence of the incubation time of cells with NPs (24 h). Specifically, when ROS was produced by the NPs, the cells started to cope with the ROS effect by modulating different signaling/stress response pathways to reduce the amount of ROS and restore homeostasis. The radiation was also independently found to induce ROS; however, the cells exposed to NPs seem to manage differently the generated damage since the NPs already influenced the cell’s response to stress.

Melanoma is considered to be radioresistant due to some specific cell characteristics such as high repair and proliferation capacity, poor cell differentiation or cancer stem cells [59]. Radiosensitivity may be however improved by using higher doses or dose rates, radiosensitizers, X-ray photodynamic therapy or inducing tumor reoxygenation by fractioned radiotherapy. In addition, one may help in triggering the antitumor immune response [60] by applying different technological strategies that could involve either fractioned or particle-assisted therapies. Thus, even though a limitation of the study is the effective applications and use of NPs on melanoma cells in patients due to their limited access to specific metastatic sites, one may predict their beneficial application to medically-oriented research that involve testing precise doses for personalized, eventually synergetic, therapies. Further investigation of the structure-function relationship, surface functionalization, cellular uptake and fate will allow developing new strategies for different therapies in cancer.

## 4. Materials and Methods

Y_2_O_3_ NPs dispersed in water at a concentration of 20% weight/volume were purchased from US research Nanomaterials, Inc. (3302 Twig Leaf Lane, Houston, TX 77084, USA; https://www.us-nano.com/) (accessed on 10 April 2021). According to the manufacturer, Y_2_O_3_ NPs are spherical and 99.99% pure, with a specific surface area of 65–85m^2^/g.

Cell culture media: DMEM high glucose (#11960044), Fetal Bovine Serum (#10270106), Penicillin-Streptomycin (#15140122), sodium pyruvate (#11360070), HEPES (#15630080) were procured from Gibco (Life Technologies, Paisley, UK).

Reagents: CellTiter 96 Aqueous One Solution Cell Proliferation Assay (MTS) was acquired from Promega (#G3582). CellROX Green (#C10444), Mitotracker Deep Red FM (#M22426), Monoclonal anti-mouse γ-H2AX (#MA1-2022) and donkey anti-mouse AlexaFlour594 (#A32744) were purchased from Invitrogen (Life Technologies, Carlsbad, CA, USA).

### 4.1. XRD and SEM

Before XRD and SEM analyses, Y_2_O_3_ NPs were dried at room temperature for 48 h. The SEM images and EDX pattern were taken with an Apreo SEM System working at a pressure of 10^−3^ Pa with acceleration voltage of 10 kV and a beam current of 20 nA. Using the image processing software (ImageJ version 1.8.0_112, National Institute of Health, Bethesda, MD, USA), the SEM image was converted from RGB to 16-bit gray scale, smooth, then adjusted for brightness/contrast in order to enhance edges of the NPs. With the “Set scale” function we convert the “Straight“ line tool from number of pixels to nanometers. Then, using this tool, we measured the size of more than 200 NPs from the image. The X-ray diffraction patterns were recorded with a Bruker D2 Phaser diffractometer equipped with a Cu-K X-ray source (λ = 1.5406 Å) and an ultra-fast LYNXEYE detector. The voltage was set at 30 kV with a current of 10 mA. The data were collected in 2ϴ geometry with the scanned angle ranging between 20° and 80°, a step of 0.01 o and 0.1 s integration time.

### 4.2. X-ray Irradiation Set-Up

The X-ray irradiation set-up is composed of an ISOVOLT Mobile 160 X-ray generator, part of a more complex computerized radiography home-made station that provides non-destructive X-ray inspections [61]. The X-ray generator can operate at maximum parameters of 160 kV with 10 mA (1600 W). All irradiation experiments were performed by keeping identical geometrical condition with the X-ray generator working at 100 kV and 8 mA and the cell cultures placed at a distance of 35 cm from the X-ray generator. The dose rate of 1.33 cGy/s was measured with a Farmer Chamber 30010 calibrated for Air Kerma (PTB-Braunschweig) connected to a Standard Unidos dosimeter. Total doses of 2, 4, and 6 Gy were delivered to cell cultures within 2 min and 30 s, 5 min and 7 min and 30 s respectively. A schematic representation of the irradiation geometry and the corresponding X-ray spectrum simulated with PENELOPE-2014 code are presented in Figure S4. More details about the geometry and simulation parameters can also be found in the Supplementary materials.

### 4.3. Cell Culture

A375 amelanotic melanoma cell line was a kind gift from the Institute of Biochemistry of Romanian Academy. The A375 cell line was cultivated in DMEM high glucose supplemented with 10% FBS and 1% penicillin/streptomycin at 37 °C and 5% CO_2_.

### 4.4. Y_2_O_3_ NPs Treatment

A375 cells at 90% confluence were harvested using 1 mL of 0.025% trypsin-EDTA for 5 min at 37 °C and counted using a Neubauer chamber. The cells were plated at different seeding densities on coverslips or directly in 24 wells plates according to the experiment being performed. Prior to incubation with NPs the cells were left to attach for 24 h. Y_2_O_3_ NPs were resuspended in complete cell culture media and diluted to the following concentrations: 100 µg/mL, 50 µg/mL, 25 µg/mL and 0 µg/mL. Cells were treated with NPs for 24 h prior to any experiment.

### 4.5. Intracellular ROS Assay

The intracellular generation of reactive oxygen species (ROS) was evaluated using the kit CellROX GREEN according to the manufacturer’s instructions. Briefly, 40,000 cells were seeded in 12-well plates for 24 h followed by another 24 h exposure to Y_2_O_3_ or by exposure to 2, 4, and 6 Gy of radiation. Afterwards the cells were washed three times with 1x PBS and subsequently stained with 100 µL of 5 µM CellROX Green for 30 min in the dark at 37 °C, 5% CO_2_. After removing the staining solution and washing with sterile 1x PBS, the cells were fixed with 4% paraformaldehyde (PFA) for 15 min at room temperature and visualized by fluorescence microscopy using an Olympus IX83 at 20× magnification.

### 4.6. DNA Double-Strand Breaks Assay

The DNA double-strand breaks can be observed by fluorescence microscopy when staining the specific histone that regulates the DNA repair at the sites of the damage. We have thus tested the capacity of Y_2_O_3_ NPs to tailor the effect of X-ray irradiation over A375 cancer cells [62].

A375 cells were cultured on coverglasses (with NPs incorporated or not) and irradiated as described earlier. Cells were fixed in 4% PFA for 15 min and permeabilized with 0.1% Triton X-100 in PBS. Cells were then blocked with 5% bovine serum albumin (BSA) for 1 h and incubated overnight at 4 °C with mouse anti-γ-H2AX antibody in 5% BSA in PBS (dilution 1:2000). Cells were washed three times with PBS and donkey anti-mouse secondary antibodies coupled with AlexaFlour-594 (dilution 1:1000) was added for 1 h at room temperature. Nucleus staining was performed using Hoescht at 1:10,000 dilution for 2 min. Finally, slides were washed with PBS, mounted, and examined using a confocal microscope ZEISS LSM 700, 63X objective (1.4 NA). Acquired images were processed using ImageJ software.

### 4.7. Clonogenic Assay

Irradiated cells were trypsinized and counted using a Neubauer chamber. One hundred cells were seeded in 35 mm Petri dishes. After at least 6 division cycles post-irradiation, cells were fixed with 4% PFA in PBS for 30 min and stained with 0.05% crystal violet in PBS for 30 min at room temperature. Colonies were counted using an optical microscope and only those formed of more than 50 cells were considered. The colony-forming data analyzed by fitting the obtained values to the linear-quadratic (LQ) model equation) are presented in Figure S1. The formula used to plot the LQ model was: Y = $e^{-\left(\alpha *D+\beta *D^{2}\right)}$; where Y is the fraction of surviving cells and D is the dose. The software used to analyze was GraphPad Prism 6. Template file for calculating the survival fractions using the LQ model is provided by GraphPad [63].

### 4.8. Mass Spectrometry Sample Preparation

Cell culture. For mass spectrometry, 1 million cells were plated in a 10 cm^2^ dish and left to adhere and reach 90% confluence. After, treatment with NPs at 50 μg/mL was made for 24 h. Next the cells were subjected to 6 Gy dose of irradiation.

Sample preparation. Sample preparation for nanoLC-MS/MS analysis was performed as previously described [64]. Briefly, proteins were extracted using chaotropic reagents, reduced, alkylated and subjected to overnight trypsin digestion. The obtained peptides were desalted on stage-tips, further concentrated to dryness in a SpeedVac and kept at −20 °C until further use.

nanoLC-MS/MS analysis. Before injection, samples were reconstituted in solvent A (0.06% formic acid-FA and 2% acetonitrile-ACN) and subjected to reversed-phase separation on a C18 analytical column (150 mm × 75 µm internal diameter, Thermo Fisher Scientific, Waltham, MA, USA) using a 2–30% solvent B (0.06% FA and 80% ACN) gradient for 240 min. The separation instrument was connected online to an LTQ-Orbitrap Velos Pro (Thermo Fisher Scientific) mass spectrometer for peptide detection. For data acquisition, a top 15 data-dependent method was used, in which an Orbitrap survey scan was followed by the fragmentation of the top 15 most abundant peptide ions.

Data analysis. Raw data was searched with the SEQUESTHT algorithm integrated into Proteome Discoverer v1.4 against the human version of the UniProt database using the following settings: trypsin as the protease used with maximum two missed cleavages, 10 ppm mass accuracy for the precursor ions and 0.5 Da for fragment ions, Met oxidation as a dynamic modification and Carbamidomethylation on Cys residues as a static modification. For control of the FDR the data was also searched against a decoy database containing the reversed sequences and the results were filtered for 1% FDR at PSM level and 5% at protein level.

Statistical analysis. Data are presented as mean ± standard error of the mean (SEM). Dependent variables were analyzed by *t*-test using GraphPad Prism, version 6. A value of *p* < 0.05 was considered significant.

## 5. Conclusions

We have evaluated the effect of Y_2_O_3_ NPs under X-ray irradiation on A375 melanoma cells. An increase of NPs concentration was found to increase intracellular ROS production. X-ray was also found to induce ROS production with increasing the dose. A colony-forming assay showed a synergic effect of NPs and radiation by evidencing the decrease of the number of proliferating cell colonies with increase of X-ray dose. DNA damage quantified by the number of foci was found in cells treated with Y_2_O_3_ NPs and exposed to various X-ray doses. The proteomic profile analysis identified a greater number of proteins involved in DNA damage response and repair for cell samples exposed to 50 µg/mL Y_2_O_3_ NPs and 6 Gy X-ray dose as compared with irradiation only. We propose that the combined approach of exposing cancer cells to both Y_2_O_3_ NPs and superficial X-ray irradiation may improve the radiotherapeutic effect.

## Figures and Tables

**Table 1:** Number of unique peptides and PSMs distributions for proteins involved in DNA damage response identified by mass spectrometry analysis.

Accession	Description	Unique Peptides	PSM-CTRL	PSM-NP	PSM-6Gy	PSM-NP+6Gy
O14757	Serine/threonine-protein kinase Chk1 OS =Homo sapiens (Human) OX = 9606 GN = CHEK1 PE = 1 SV = 2 − [CHK1_HUMAN]	1	4	3	5	1
P38936	Cyclin-dependent kinase inhibitor 1 OS = Homo sapiens (Human) OX = 9606 GN=CDKN1A PE = 1 SV = 3 − [CDN1A_HUMAN]	3	1	6	NA	5
P11802	Cyclin-dependent kinase 4 OS = Homo sapiens (Human) OX = 9606 GN = CDK4 PE = 1 SV = 2 − [CDK4_HUMAN]	2	1	3	1	5
Q00534	Cyclin-dependent kinase 6 OS = Homo sapiens (Human) OX = 9606 GN = CDK6 PE = 1 SV = 1 − [CDK6_HUMAN]	6	13	9	9	21
P13010	X-ray repair cross-complementing protein 5 OS = Homo sapiens (Human) OX = 9606 GN = XRCC5 PE = 1 SV = 3 − [XRCC5_HUMAN]	41	78	94	73	86
P12956	X-ray repair cross-complementing protein 6 OS = Homo sapiens (Human) OX = 9606 GN = XRCC6 PE = 1 SV = 2 − [XRCC6_HUMAN]	51	130	139	139	155
P49959	Double-strand break repair protein MRE11 OS = Homo sapiens (Human) OX = 9606 GN = MRE11 PE = 1 SV = 3 − [MRE11_HUMAN]	9	9	16	10	16
Q92878	DNA repair protein RAD50 OS = Homo sapiens (Human) OX = 9606 GN = RAD50 PE = 1 SV = 1 − [RAD50_HUMAN]	6	9	11	7	4

## Data Availability

Not applicable.

## Supplementary Materials

The following are available online. Figure S1. Clonogenic assay of samples treated with 0, 25, 50, 100 μg/mL Y_2_O_3_ and exposed to 0, 2, 4, 6 Gy doses of radiation (by fitting the obtained values to the linear-quadratic model equation); Figure S2. Total, Compton and photoelectric mass attenuation coefficients for Y_2_O_3_, total mass attenuation for water and simulated X-ray spectrum with PENELOPE-2014 code; Figure S3. Viability of A375 cells by MTS assay, after treated with different concentrations of Y_2_O_3_ nanoparticles. (* *p* value < 0.005, n = 3 ± SEM). The cells were monitored at two time points: 8 h and 24 h; Figure S4. Schematic representation of the irradiation geometry and the associated X-ray spectrum generated with PENELOPE-2014 code.
