# Supplementary material for: Y2O3 Nanoparticles and X-ray Radiation-Induced Effects in Melanoma Cells
Source: Molecules. 2021 Jun 4;26(11):3403. doi: 10.3390/molecules26113403 (PMC8200002; doi:10.3390/molecules26113403)
Supplement: Supplementary file 1 [file molecules-26-03403-s001.zip › molecules-1244101-supplementary.pdf]

molecules MDPI

Supplementary materials

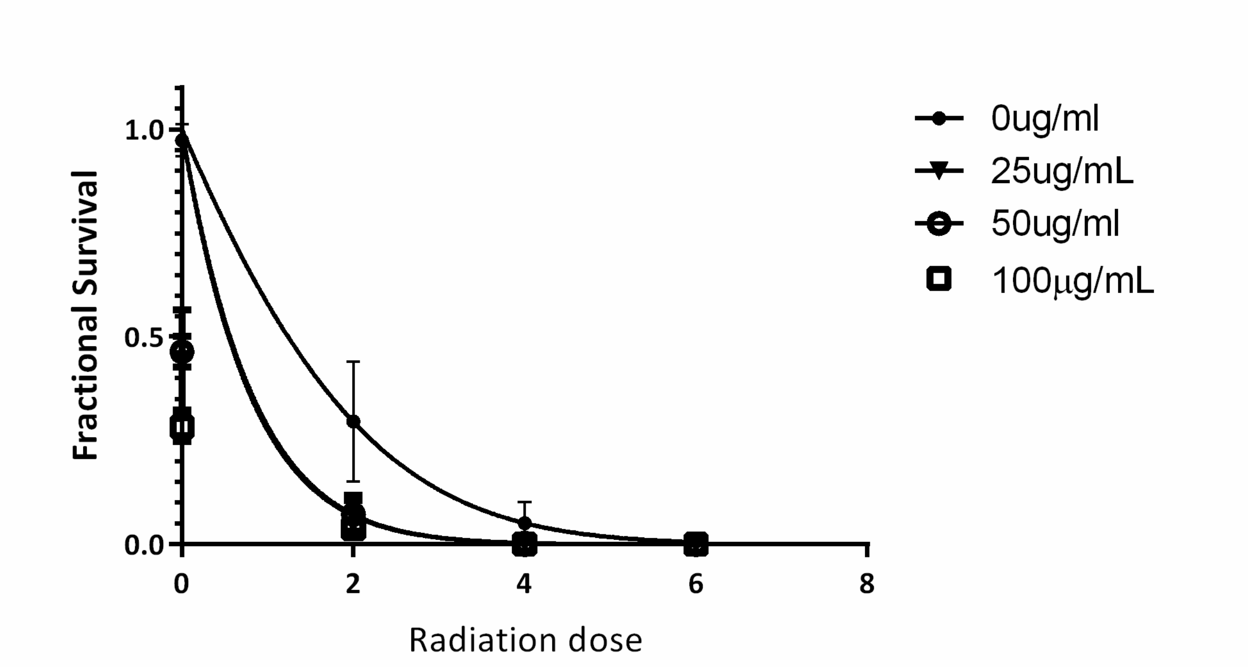

Figure S1. Clonogenic assay of samples treated with 0, 25, 50, 100 µg/mL Y_2O_3 and exposed to 0, 2, 4, 6 Gy doses of radiation (by fitting the obtained values to the linear-quadratic model equation).

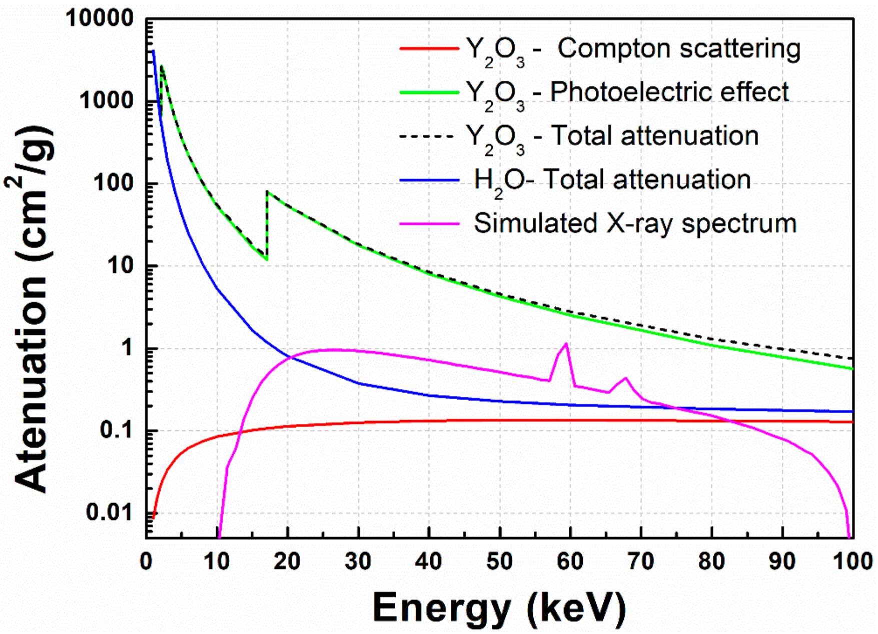

Figure S2. Total, Compton and photoelectric mass attenuation coefficients for Y_2O_3, total mass attenuation for water [1] and simulated X-ray spectrum with PENELOPE-2014 code [2].

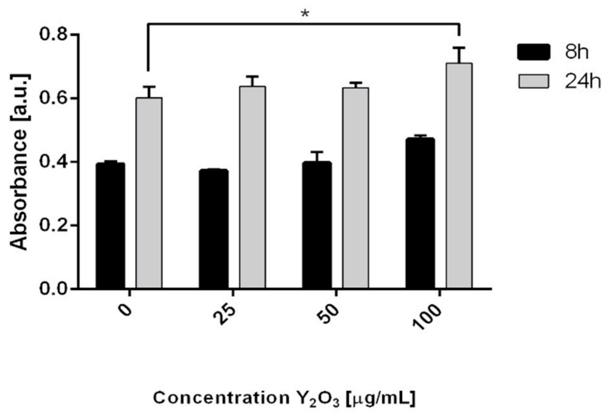

Figure S3. Viability of A375 cells by MTS assay, after treated with different concentrations of Y_2O_3 nanoparticles. (* p value<0.005, n=3±SEM). The cells were monitored at two time points: 8h and 24h

The CellTiter 96 AQueous One Solution Proliferation Assay (MTS) was performed in a 24well plate according to the manufacturer's guidelines. Briefly, 40,000 cells were seeded and left to attach for 24h. The Y_2O_3 NPs were incubated at 0, 25, 50 and 100µg/mL concentration in complete cell culture media for 24h. Afterwards, cells were washed twice with sterile 1x PBS (Phosphate buffered saline-13mM NaCl, 2.7 mM KCl, 10 mM Na2HPO4, 1.8 mM KH2PO4) 3 times and a mix containing 500µL complete culture media and 100µL CellTiter 96 AQueous One Solution Reagent was added per sample and left to incubate at 37°C. After 30min, 100µL of the mix was moved to a 96well plate and the absorbance at 490nm was read using Apollo LB913 UV-VIS spectrophotometer.

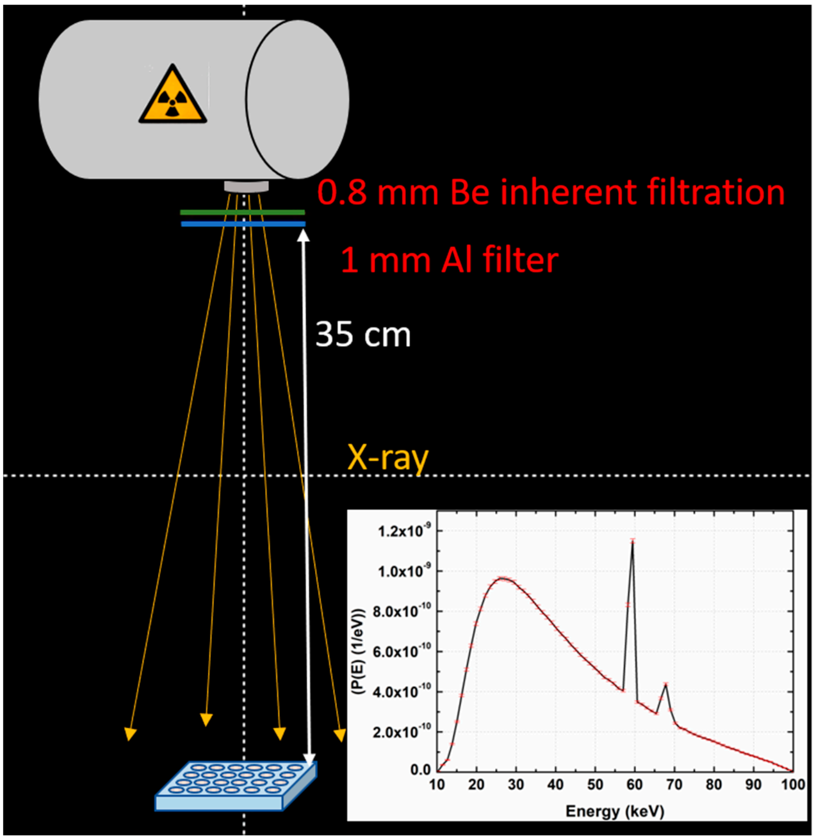

Figure S3. Schematic representation of the irradiation geometry and the associated X-ray spectrum generated with PENELOPE-2014 code [2].

Monte Carlo simulation geometry and details

The representation of the irradiation setup is presented in figure Figure S2. The X-ray spectra inserted in the figure was generated with PENELOPE-2014 code [2]. The simulation starts with a monoenergetic electron beam of 100 keV hitting a tungsten anode. The produced X-ray photon are then transported through a Be filter of 0.8 mm, an Al foil of 1 mm and detected in a water phantom of 3 mm thickness placed at 35 cm from the Al filter as in the experimental condition. The cutoff energy of electron photon and positron (EABS1:3) transport trough each simulated material was set at 5 keV, 1 keV and respectively 5 keV. The parameters for the elastic scattering was set as C1= C2 =0.05 and the cutoff for bremsstrahlung emission and inelastic collisions were set at W_{CR} =W_{CC} =2 kev. As variance reduction technique we use the interaction forcing for electrons with a forcer factor of 400 for both hard bremsstrahlung emission (ICOL=4) and inner shell impact ionization (ICOL=5). Also, we used bremsstrahlung and X-ray splitting with a factor of 2 for tungsten anode , Be and Al filter. A total of $1x10^8$ histories were simulated.

References:

1. https://www.physics.nist.gov/PhysRefData/Xcom/html/xcom1.html.
2. Salvat, F. *PENELOPE-2014: A code system for Monte Carlo simulation of electron and photon transport*; NUCLEAR ENERGY AGENCY, Organisation for Economic Co-operation and Development, Barcelona, Spain, 2015; Volume NEA/NSC/DOC(2015)3, p. 386.
